# Rabies Surveillance in Mainland Tanzania: A Scoping Review of Animal Rabies Occurrences (1993–2023)

**DOI:** 10.3390/pathogens14090919

**Published:** 2025-09-11

**Authors:** Emmanuel Kulwa Bunuma, Julius Keyyu, Joseph Maziku, Stella Bitanyi, Robert Fyumagwa, Katendi Changula, Benjamin Mubemba, Edgar Simulundu, Simbarashe Chitanga, Daniel L. Horton, Abel Bulamu Ekiri, Hirofumi Sawa, Walter Muleya

**Affiliations:** 1 Department of Biomedical Sciences, School of Veterinary Medicine, The University of Zambia, Lusaka P.O. Box 32379, Zambia; emmanuelbunuma@gmail.com; 2 Tanzania Veterinary Laboratory Agency (TVLA), Dar es Salaam P.O. Box 9254, Tanzania; nstelbi@yahoo.co.uk; 3 Tanzania Wildlife Research Institute (TAWIRI), Arusha P.O. Box 661, Tanzania; julius.keyyu@tawiri.or.tz; 4 Commission for Science and Technology (COSTECH), Dar es Salaam P.O. Box 4302, Tanzania; joseph.maziku@costech.or.tz; 5 Wildlife Conservation Initiative (WCI), Arusha P.O. Box 16020, Tanzania; robert.fyumagwa@gmail.com; 6 Department of Paraclinical Studies, School of Veterinary Medicine, University of Zambia, Lusaka P.O. Box 32379, Zambia; katendi.changula@gmail.com; 7 Department of Wildlife Sciences, School of Natural Resources, Copperbelt University, Ndola 50100, Zambia; mubembab85@yahoo.co.uk; 8 Macha Research Trust, Choma P.O. Box 630166, Zambia; edgar.simulundu@macharesearch.org; 9 Department of Preclinical Studies, School of Veterinary Medicine, University of Namibia, Windhoek 13301, Namibia; schitanga@gmail.com; 10 Department of Comparative Biomedical Sciences, School of Veterinary Medicine, University of Surrey, Guildford GU2 7XH, UK; d.horton@surrey.ac.uk (D.L.H.); ab.ekiri@surrey.ac.uk (A.B.E.); 11 Institute for Vaccine Research and Development, Hokkaido University, Sapporo 001-0021, Japan; h-sawa@ivred.hokudai.ac.jp; 12 One Health Research Center, Hokkaido University, Sapporo 001-0020, Japan; 13 International Institute for Zoonosis Control, Hokkaido University, Sapporo 001-0020, Japan; 14 Department of Disease Control, School of Veterinary Medicine, The University of Zambia, Lusaka 10101, Zambia; 15 Global Virus Network, Baltimore, MD 21201, USA

**Keywords:** Tanzania, *Lyssaviruses*, rabies hotspots, wildlife reservoirs, passive surveillance

## Abstract

Animal rabies remains underreported in low-income countries, hindering effective control. This scoping review aimed to map reported animal rabies cases, identify key reservoir species, and assess gaps in surveillance coverage in mainland Tanzania from 1993 to 2023. Specifically, it addressed the distribution of cases, species involved, and the extent of surveillance coverage during this period. Literature searches in PubMed, Google Scholar, and Science Direct were screened using Rayyan. Twenty articles published between 1993 and 2023 reported 7319 animal rabies cases across the Northern Zone (NZ), Southeastern Zone (SEZ), and Coastal Zone (CZ). In the NZ, domestic dogs accounted for most cases (5387), followed by jackals (225), cats (77), livestock (311), and various wildlife species including African wild dogs, bat-eared foxes, lions, cheetahs, and striped hyenas. Additionally, 102 cases involved unidentified animals. In SEZ, domestic dogs (588) were the primary source, followed by jackals (262), hyenas (8), cats (10), honey badgers (5), and leopards (2). In CZ, domestic dogs accounted for 94 cases. The findings confirm domestic dogs as the main rabies reservoir, highlighting the need for strengthened surveillance and control. The role of wildlife in rabies maintenance and spillover remains poorly understood and warrants further investigation, especially in enzootic hotspots.

## 1. Introduction

Rabies is a fatal zoonotic neurological disease caused by the *Lyssavirus* genus, which are RNA viruses in the family *Rhabdoviridae* that infect mammals worldwide [1], and are capable of infecting all mammals [2,3]. Annually, it accounts for an estimated 59,000 human deaths worldwide, with over 98% of these fatalities linked to canine rabies [4]. Despite its impact, the current passive surveillance data on canine rabies, especially in regions with the highest burden, are insufficient to support informed decision-making for effective control efforts [5,6]. Key gaps include a lack of official reporting regarding the incidence of and exposure to animal rabies [7]. This has been demonstrated by certain active *lyssavirus* biosurveillance efforts, which have revealed significant discrepancies between officially reported figures and actual rabies-related deaths, which all show much higher mortality than officially reported [6,8]; for example, contact tracing in northern Tanzania found that at least 20% of rabies exposures and deaths were not recorded in official sources, highlighting significant underreporting according to Hampson et al. (2008) [9].

Reliable estimates of the domestic dog population in Tanzania are also lacking [10]. Local surveys suggest that the actual numbers may be substantially higher than official records. For example, Gsell et al., (2012) [11] recorded 2498 dogs in 7993 households in Iringa town, far more than local estimates, exacerbating the challenge of low vaccination coverage and the resulting public health risk [12]. Wildlife species like jackals have also been linked to rabies transmission. They may act as both dead-end hosts and contributors to maintaining the virus in the ecosystem [13]. This further amplifies the public health threat, especially in areas where domestic dog vaccination is inadequate [14,15]. However, the role of wildlife in rabies epidemiology across Tanzania remains poorly understood, largely due to inadequate infrastructure for the biosurveillance of viruses within the *Lyssavirus* genus.

Studies have shown that clinical signs can be reliable indicators of rabies where laboratory confirmation is limited [12,16]. For example, over 75% of suspected rabies cases have been confirmed positive after biological testing, highlighting the usefulness of clinical and epidemiological criteria [17].

Surveillance studies backed by reliable data have been instrumental in enhancing control and prevention efforts. For example, Knobel et al. (2008) [18] estimated that canine rabies was responsible for approximately 55,000 human deaths each year across Africa and Asia. This has led to intensified global efforts to eliminate rabies, with a target of zero human deaths by 2030. Therefore, active surveillance in animals will provide a comprehensive understanding of the circulating genotypes and lyssavirus diversity in Tanzania. It will also serve as an early warning system for potential outbreaks, particularly in areas bordering national parks and forested regions where spillover from the sylvatic cycle is possible [19].

A scoping review was selected due to the wide variation in study designs and reporting, enabling the comprehensive mapping of evidence and gaps in rabies surveillance and reporting in Tanzania, particularly for domestic and wild animal hosts. Due to persistent underreporting, limited laboratory confirmation, and unclear wildlife reservoir dynamics, a synthesis of existing evidence is needed. Accordingly, this scoping review addresses the following research question: What is the distribution, species involvement, and surveillance coverage of animal rabies in mainland Tanzania from 1993 to 2023?

## 2. Methods

### 2.1. Data Source and Search Strategy

This review followed the Preferred Reporting Items for Systematic Reviews and Meta-Analyses Extension for Scoping Reviews (PRISMA-ScR) guidelines [20] to assess existing studies on animal rabies cases, providing a foundation for more comprehensive systematic analyses [21]. A completed PRISMA-ScR checklist is provided in the Supplementary Materials (Supplementary Table S1), and no review protocol was registered. The articles were retrieved from three databases: PubMed, Google Scholar and Science Direct. The keywords (“Rabies”) OR (“Lyssavirus”) AND (“Tanzania”) were used to search for articles published between 1 July 1993 and 12 September 2023. Searches were performed on 7 July 2023, and 12 September 2023. The results were exported to the screening tool Rayyan [22].

### 2.2. Inclusion and Exclusion Criteria

Original peer-reviewed articles were eligible for review if they met the following criteria: (1) published between 1993 and 2023, (2) original research articles, and (3) written in English. We included studies reporting rabies cases in animals (wildlife, livestock, and canines), where diagnosis was based on either clinical signs or laboratory testing conducted in mainland Tanzania. On the other hand, articles that were non-original in nature, such as conference proceedings, perspectives, commentaries, opinions, reports, letters, meta-analyses, duplicate studies, and those without full-text access, were excluded. The review period from 1993 to 2023 was selected to include the earliest records of animal rabies cases published in peer-reviewed journals and to pinpoint the geographic regions with the highest occurrence rates. All retrieved articles were screened by title and abstract in Rayyan. Full texts of potentially eligible studies were then retrieved and assessed for eligibility according to the predefined criteria. Duplicate records were removed using Rayyan’s duplicate detection feature. Clinical signs consistent with rabies were defined according to WHO guidelines: sudden behavioral changes, aggression, paralysis, excessive salivation, and progressive neurological dysfunction.

### 2.3. Data Extraction and Synthesis

Data extraction was conducted using a simple standardized excel sheet developed before the review. To enhance reliability, data selection and extraction were conducted by one reviewer and independently cross-checked by a second reviewer. For all studies selected at the abstract level, data were extracted and presented in a table. We recorded the year and location of each study. For studies focused on animal rabies, the following information was retrieved: the source of the sample or clinical signs used for diagnosis, historical and epidemiological data, the type of diagnostic test conducted, laboratory test results, the species affected, the lyssavirus species or rabies virus (RABV) variant in circulation, and the study location. For studies reporting dog bite occurrences, we extracted details on the location, whether the bite was followed up, and the confirmation of rabies in the dog through clinical signs or laboratory tests. Due to the heterogeneity of the studies, a narrative synthesis was performed.

## 3. Results

### 3.1. Study Selection

Comprehensive literature searches were performed across Science Direct (64), PubMed (129), and Google Scholar (195), respectively. After removing 197 duplicates, 191 records remained for title screening. Titles and abstracts were independently screened based on the review questions, leading to the exclusion of 156 articles. Full texts of the remaining 35 articles were retrieved to assess eligibility.

**Figure 1:**
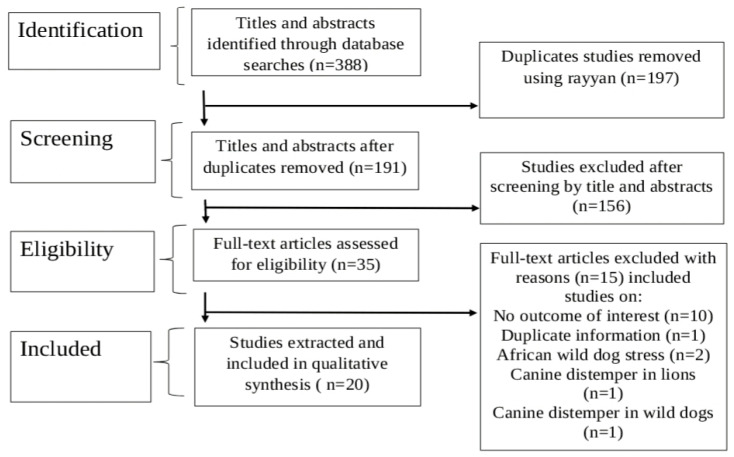
PRISMA flow diagram that includes searches of databases.

Ultimately, 20 eligible studies (detailed in Supplementary Table S2) were selected for data extraction and synthesis in this review (Figure 1), with a summary provided in Table 1.

### 3.2. Critical Appraisal of Sources

The methodological quality of included studies was not evaluated due to the scoping nature of the review; however, future reviews could apply a formal risk of bias tool such as ROBINS-I to strengthen the methodological rigor [42,43].

### 3.3. Characteristics of the Studies Included in the Review

Table 1 summarizes key attributes across the 20 quantitative studies reviewed. Based on Tanzanian administrative zones [44], our study categorized surveillance cases into three zones: The Northern Zone, which includes Arusha, Kilimanjaro, Tanga, and Mara; the Southern Eastern Zone, comprising Morogoro, Iringa, Njombe, Ruvuma, Lindi, and Mtwara; and the Coastal Zone, represented by Dar es Salaam.

Most studies (14 out of 20) focused on Northern Tanzania, particularly in the Serengeti National Park and Ngorongoro Conservation Area. The Serengeti National Park, adjacent to Kenya’s Maasai Mara National Park, allows cross-border wildlife movement, enhancing the scope for rabies transmission studies. These studies span from 1993 to 2023. Only one study conducted within the past 30 years involved 25 free-tailed bats (*C. pumilus*) sampled within the Serengeti National Park; this was a serosurveillance study yielding negative results [33]. Another study identified a unique lyssavirus, IKOV, in an African civet [32]. The remaining studies examined other host species, including cattle, goats, and dogs, all of which tested positive for rabies virus (RABV) [28].

Among the 20 studies, nine were retrospective cohort studies (Table 1) analyzing previously collected data or samples to investigate past rabies occurrences in Tanzania. The lyssavirus variants identified in circulation include the canine rabies variant, as well as an assumed bat rabies variant, for which the reservoir remains unidentified. All studies relied on passive surveillance, except for one that employed active surveillance. The diagnostic methods ranged from clinical signs and serosurveillance to advanced molecular techniques. Starting in the 1990s, fluorescent antibody tests were the primary diagnostic tool, targeting domestic dogs and wildlife. Between 2000 and 2010, molecular techniques such as gene sequencing and nucleoprotein sequencing were introduced, expanding host surveillance to include species like hyenas, mongooses, and badgers. From 2011 to 2020, advanced genomic methods, including direct rapid immunohistochemical testing (dRIT) and genomic sequencing, became standard, with a continued emphasis on domestic dogs and wildlife reservoirs. During 2021–2023, genomic sequencing was extensively applied to archived samples, particularly those collected from Southern Eastern Zone.

### 3.4. Reservoir Hosts and Their Role in the Circulation of Lyssavirus in Tanzania

Our review found that both wildlife and domestic dogs can sustain transmission chains and possess the capacity for persistence [17,25,26]. The following animals were identified in various studies as important in rabies transmission. Domestic dogs were identified as the primary reservoir hosts, playing a crucial role in rabies persistence, especially in the Northern Zone and to a small extent in the Southern Zone [10,26], while no dogs breeds were mentioned in all the 20 studies (Table 2). Rabies cases have been reported in wildlife such as jackals and hyenas, which are considered potential reservoir hosts capable of sustaining transmission in both the Northern and Southeastern Zones [38]. However, no bat species have been identified as a reservoir to date.

### 3.5. Rabies Hotspots in Tanzania Mainland (1993–2023)

**Figure 2:**
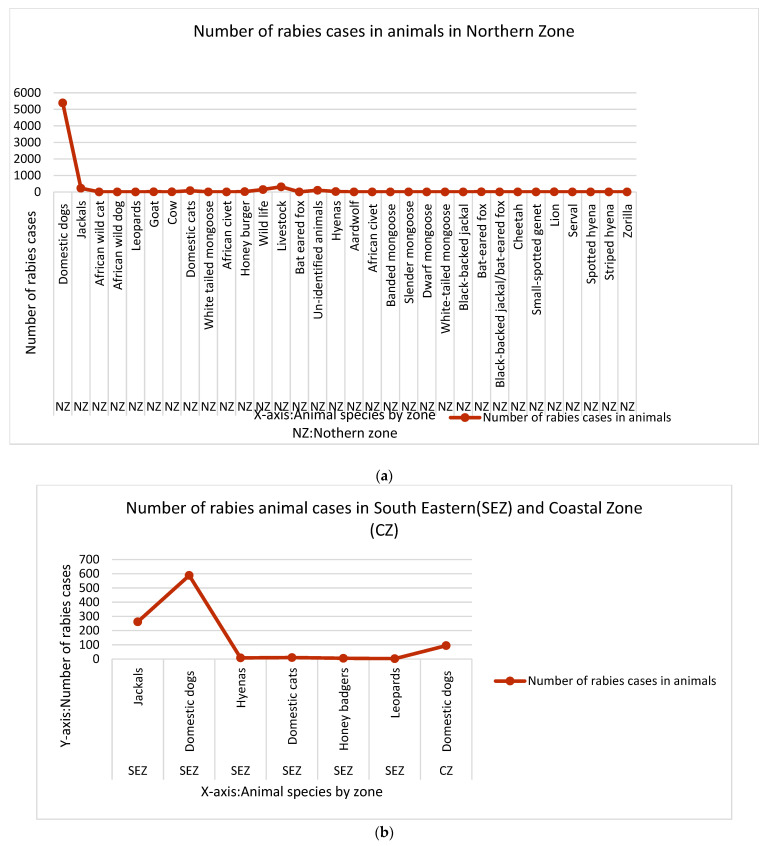
(**a**) Reported and published animal rabies cases in Tanzania (1993–2023) by species and zone. (**b**) Reported and published animal rabies cases in Tanzania (1993–2023) by species and zone.

The occurrence of rabies cases in Tanzania was unevenly distributed, with higher concentrations observed in Northern Tanzania, followed by southeastern regions; due to a lack of surveillance there could be other hotspots, yet we have no data to determine the occurrences instead of the absence of rabies. Northern Tanzania (NT) reported the highest rabies burden, with 5387 cases in domestic dogs [26,27,29], 225 in jackals, 145 in wildlife [40], 311 in livestock and 2 in white-tailed mongoose [30] (Figure 2a,b). In Southern Eastern Tanzania (SET), rabies cases were also significant, especially among domestic dogs (588 cases) and jackals (252 cases), along with eight hyenas [32,34,39], highlighting the region’s notable wildlife rabies burden.

The Coast Region (CR) saw fewer cases, with these mainly affecting domestic dogs, with 94 reported cases [37]. Overall, rabies transmission appeared enzootic in the identified hotspots, with domestic dogs, jackals, and wildlife species being the most affected, particularly in the Northern and Southeastern Zones. Additionally, 102 cases involved unidentified species [40].

## 4. Discussion

The endemicity and neglect of rabies in mainland Tanzania have been exacerbated by the lack of reliable surveillance case statistics [10,15]. This scoping review revealed that animal rabies surveillance in Tanzania relies mainly on a passive, laboratory-based system managed by the Ministry of Livestock and Fisheries Development, in collaboration with the Tanzania Veterinary Laboratory Agency, Tanzania Wildlife Research Institute and local university laboratories. These institutions perform standard diagnostic tests, such as the direct fluorescent antibody test and genomic surveillance, which collectively form the core of the national rabies monitoring framework and covers nearly all animal samples tested for rabies in Tanzania. However, because passive surveillance depends on voluntary reporting and limited diagnostic capacity, many cases likely go undetected, leading to an underestimation of the true burden of rabies [45]. This underreporting reduces the accuracy of national statistics and makes it harder to allocate resources effectively or plan targeted interventions. Despite the ongoing national monitoring efforts by these institutions, this review has highlighted the significant lack of coordinated surveillance efforts, resulting in limited statistics, knowledge and understanding of definitive host reservoirs in the reported ecosystems.

Research on rabies has predominantly focused on humans and dogs, while rabies in livestock and wildlife has received significantly less attention [46]. As a result, there is likely limited information and understanding of the epidemiology, transmission dynamics, and impact of rabies in livestock and wildlife compared to what is known about rabies in humans and dogs. Undetected and underreported animal rabies cases not only increase the risk of human exposure but also impose significant economic costs on households. Affected families may spend large sums on post-exposure prophylaxis (PEP), livestock replacement, or travel for treatment. Although lyssavirus species other than the classical rabies virus (RABV), such as Ikoma virus (IKOV), have been identified in Tanzania, the role of wildlife in rabies transmission remains poorly understood. The primary strain responsible for most human and animal cases continues to be the classical RABV, which is transmitted predominantly by domestic dogs [30]. In Tanzania [13], most livestock rabies cases are attributed to bites from rabid dogs, highlighting the critical role of canine rabies control in reducing livestock infections. While livestock are typically considered dead-end hosts for rabies, rare cases of zoonotic transmission from livestock to humans have been documented [46].

This review found that domestic dogs accounted for most reported rabies cases among animals in Tanzania from 1993 to 2023. Rabies surveillance during this period was predominantly passive, relying heavily on human–animal interactions and potential human exposure to the virus from animal reservoirs [7]. This reliance on reported exposures, rather than the proactive monitoring of rabies in animal populations, limits our understanding of rabies transmission dynamics and hampers the development of effective control measures. Consequently, the incidence and prevalence of rabies across various species in mainland Tanzania remain poorly characterised. Moreover, lyssavirus variant typing was not available for the majority of reported rabid animals during this period. Instead, variants associated with terrestrial rabies cases were inferred based on geographic location. For instance, rabid dogs or wild terrestrial mammals were typically assumed to be infected with the canid-associated variant Africa 1b, presumed to have originated from domestic dogs or other wild carnivores [26,28]. The sole exception was a single African civet case, which was linked to *Ikoma lyssavirus* [32].

We attribute the likely underestimation of rabies cases in dogs to several key factors. First, the reported low vaccination rates among dogs [47], consistent with previous findings, highlight a significant gap in dog owners’ education about rabies prevention and the importance of maintaining animal health [46]. Additionally, achieving and sustaining the recommended vaccination coverage of 70% remains challenging, with most studies reporting vaccination rates of only around 50% [48]. This low coverage reflects limitations in how vaccination campaigns are planned and implemented. Currently, dog rabies vaccination campaigns in Tanzania are confined to a few state capitals, primarily occurring during annual World Rabies Day events or as reactive measures during outbreaks [47].

Studies in sub-Saharan Africa suggest that cost remains a major obstacle to dog vaccination [48]. This challenge is further compounded by the common practice of allowing dogs to roam freely, which complicates efforts to reach animals during vaccination campaigns. Together, these factors contribute to the significant underestimation of rabies cases in dogs. Enhancing active surveillance systems is critical to better understand rabies prevalence and to design more targeted and effective intervention strategies [27]. Practical strategies to improve vaccination coverage could include regular mass dog vaccination campaigns, engaging community animal health workers, integrating rabies vaccination with other veterinary outreach services, and strengthening cross-sectoral collaboration through a One Health approach [49]. These strategies are already under way in Tanzania, in collaboration with the World Organisation for Animal Health (WOAH) and other partners, towards achieving zero human deaths from dog-mediated rabies by 2030.

The geographical heterogeneity of rabies cases in Tanzania was evident in the uneven distribution across regions and animal species. Domestic dogs accounted for the majority of reported cases, with over 5000 cases in the Northern Zone (NZ) alone, underscoring their critical role in rabies persistence within the Serengeti ecosystem [27]. Their high population density, ability to sustain within-species transmission, and frequent spillover to other species made them the primary focus for effective rabies control [27,48]. In contrast, wildlife cases, such as those in African wildcats, leopards, and mongooses, were significantly lower [29], likely due to underreporting or limited surveillance, suggesting that the persistence of rabies in wildlife may have been greater than previously recognized. The South Eastern Zone (SEZ) and the Coastal Zone (CZ) reported fewer cases, possibly due to inadequate surveillance efforts [39,46]. Most wildlife cases were confined to NZ, reflecting potential biases in reporting. The Northern Zone stood out as a hotspot, with enzootic transmission among wildlife and spillover to other species, as evidenced by a rabid hyena in the Serengeti. Although this review is descriptive, it provides insights that can guide targeted vaccination, better wildlife monitoring, and future research on local risk factors to strengthen rabies control programs and policy [49,50]. These disparities highlight the urgent need for enhanced surveillance and targeted control strategies, particularly in high-risk areas like the Serengeti ecosystem.

Rabid bats have never been reported in Tanzania and previous studies on bats and rabies in the Serengeti, Tanzania, have consistently found negative results. For instance, *lyssavirus* screening of *Chaerephon pumilus* from the Serengeti was negative [33]. Similarly, a study that screened 3000 apparently healthy bats across sub-Saharan Africa, including Kenya, found no active lyssavirus infections [51]. It remains unclear in Tanzania whether the epizootiology and phylogenetics of rabies in bats differ from those of terrestrial rabies maintained by mammalian carnivores. Knowledge about the circulation of rabies virus variants in bat species is less developed compared to the understanding of variants found in carnivores [52]. As a result, potential rabies exposure involving bats is poorly understood, and public awareness of the risk of rabies transmission from bats remains low [53]. Alternatively, addressing this gap in understanding bats’ role in rabies transmission could involve the active genetic typing of confirmed rabies cases in other hosts. Such typing could clarify whether bats contribute to rabies transmission and enhance our understanding of rabies origins and transmission patterns, distinguishing strains maintained by domestic dogs from those potentially carried by wildlife like jackals. Improved knowledge of variant circulation would allow for more refined control measures and better strategies to protect human health [54].

This review should be interpreted with several limitations in mind. First, the heavy reliance on passive surveillance and the clustering of studies in specific zones, particularly the Northern Zone, introduce geographical bias that may distort our understanding of true rabies hotspots. Areas with limited diagnostic capacity or minimal reporting may falsely appear low-risk, masking ongoing transmission. Second, the limited genomic surveillance and the restricted geographic and temporal scope of available studies (1993–2023) further hinder a comprehensive quantitative analysis of rabies epidemiology and viral circulation across Tanzania. Third, incomplete data on key animal demographics, such as breed, sex, age and population characteristics, constrained our ability to analyze reservoir dynamics in detail. To generate more reliable estimates and inform targeted interventions, future research should prioritize active surveillance using standardized protocols, expand the genomic typing of rabies virus strains, and ensure broader geographic coverage, especially in underrepresented regions.

## 5. Conclusions

In summary, this scoping review highlights the persistence of rabies among domestic dogs and the neglected role of livestock and wildlife reservoirs in Tanzania between 1993 and 2023. To advance rabies control efforts, we recommend enhancing mass dog vaccination coverage to interrupt transmission at its primary source. Implementing active, coordinated surveillance systems, including the routine genomic typing of rabies virus in wildlife and livestock, will strengthen the early detection and understanding of potential spillover events. Finally, fostering inter-sectoral collaboration through a robust One Health approach is essential to align veterinary, public health, wildlife, and community stakeholders, ensuring sustainable and effective rabies prevention and control nationwide.

## Figures and Tables

**Table 1:** Description of selected studies included in the review (*n* = 20).

Study Authors	Tanzania Administrative Zones (s)	Type of Rabies Diagnosis Performed	Type of Surveillance	Host Animals, Sample Types, and Related Clinical/Epidemiological Data	Assumed *Lyssavirus* Variant in Circulation
Gascoyn et al., 1993 [23]	Northern Zone	Sero-surveillance	Passive surveillance	African wild dogs serum samples	Canine RABV variant
Cleaveland and Dye 1995 [24]	Northern Zone	Fluorescent antibody test, Sero-surveillance and Clinical signs observation	Passive surveillance	Rabies case records (1977–1994) and diagnostic brain and saliva samples were collected from domestic dogs, cows, bat-eared foxes (*Otocyon megalotis*), and African wild dogs (*Lycaon pictus*).	Canine RABV variant
Cleveland., 1999 [25]	Northern Zone	Sero-surveillance	Passive surveillance	Domestic dogs serum	Canine RABV variant
East et al., 2001 [26] and Lushashi et al, 2020 [27]	Northern Zone	Sero-surveillance, Clinical signs observation and Molecular screening	Passive surveillance	Samples (brain, tissue, serum, saliva) collected in 1999 from spotted hyena, bat-eared fox, black-backed jackal, white-tailed mongoose, banded mongoose, slender mongoose, and dwarf mongoose.	Canine RABV variant
Lembo et al., 2006 [28]	Northern Zone	Direct rapid immunohistochemical test (dRIT)	Passive surveillance	Brainstem samples from domestic dogs, cats, cows, goats, white-tailed mongooses, black-backed jackals/bat-eared foxes, small-spotted genets, and spotted hyenas were collected between 2002 and 2004.	Canine RABV variant
Lembo et al., 2007 [29]	Northern Zone	Gene sequencing	Passive surveillance	Archived brain samples from domestic dogs collected between 1994 and 2001.	Canine RABV variant
Lembo et al., 2008 [30]	Northern Zone	Clinical and epidemiological history, Fluorescent antibody test, Inoculation of murine neuroblastoma cells and mouse inoculation and nucleoprotein gene sequencing	Passive and active surveillance	Archived brain samples from domestic cats, livestock (cows, goats), spotted hyenas, honey badgers, African wildcats, white-tailed mongooses, bat-eared foxes, small-spotted genets, jackals, and leopards tested by 2001, with further tests until 2006.	Canine RABV variant
Swai et al., 2010 [31]	Northern Zone	Fluorescent Antibody Technique test	Passive surveillance	Archived samples from domestic dogs, jackals, and hyenas collected between 1993 and 2002	Canine RABV variant
Marston et al., 2012 [32]	Northern Zone	Gene sequencing	Passive surveillance	Archived brain samples collected from an African civet.	Assumed to be the Bat rabies variant
Horton et al., 2014 [33]	Northern Zone	Sero-surveillance	Passive surveillance	Serum bats samples	N/A
Brunker et al., 2015 [34]	Northern Zone	Gene sequencing	Passive surveillance	Archived brain samples from domestic dogs collected between 2003–2012	Canine RABV variant
Mpolya. et al., 2017 [35]	Southern Eastern Zone	Clinical signs and Fluorescent antibody test	Passive surveillance	Rabid host data: clinical, historical, and epidemiological	Canine RABV variant
Brunker et al.,2018 [36]	Northern Zone	Genomic sequencing	Passive surveillance	Archived brain samples from domestic dogs collected between 2004 and 2013.	Canine RABV variant
Mtui-Malamsha et al., 2019 [37]	Coastal Zone	Sero-surveillance	Passive surveillance	Saliva and serum from randomly selected domestic dogs	Canine RABV variant
Kirstyn et al., 2020 [38]	Northern Zone	Nucleoprotein gene sequencing	Passive surveillance	Archived brain samples from domestic dogs, livestock, and wildlife collected between 2017 and 2019.	Canine RABV variant
Lushasi et al., 2020 [27]	Southern Eastern Zone	Clinical signs and epidemiological history	Passive surveillance	Rabid host data from domestic dogs: clinical signs, biting history, and epidemiology.	Canine RABV variant
Lushashi et al., 2021 [13]	Southern Eastern Zone	Clinical signs, epidemiological history and Fluorescent antibody test	Active surveillance	Rabid host data: Cats, domestic dogs, jackals, honey badger (*Mellivora capensis*), hyena, and leopard (*Panthera pardus*) based on clinical, historical, and epidemiological records.	Canine RABV variant
Hayes et al., 2022 [39]	Southern Eastern Zone	Clinical signs and Fluorescent antibody test	Passive surveillance	From 2011 to 2018, suspected rabies cases in domestic dogs and jackals were documented.	Canine RABV variant
Mancy et al., 2022 [40]	Northern Zone	Fluorescent antibody test, gene sequencing and Clinical signs	Passive surveillance	Rabid domestic dog data: diagnosis, clinical signs, history, and epidemiology.	Canine RABV variant
Bautista et al., 2023 [41]	Southern Eastern Zone	Genomic sequencing	Passive surveillance	Domestic dogs Samples archived from 2021	Canine RABV variant

N/A: Not applicable, RABV: Rabies virus.

**Table 2:** The roles of various hosts in *Lyssavirus* transmission across different geographical zones in Tanzania.

Host Type	Examples of Species	Role in Rabies Ecology	Relationship to Transmission Dynamics	Hotspot Zones
Reservoir (Primary host)	Domestic dogs	Main reservoir sustaining rabies transmission	Key source of transmission to incidental hosts and wildlife	Serengeti, Southeast Zone
Potential Reservoirs	Jackals, Hyenas, African wild dogs, Bats	Possible secondary reservoirs (uncertain role)	Facilitate spillover and occasional spillback to domestic dogs	Serengeti, Southeast Zone
Incidental Hosts	Domestic cats, Livestock (Cows, Goats), Wild carnivores (Honey badgers, Mongooses), Humans	Do not sustain the virus; acquire rabies through contact with reservoirs or potential reservoirs	Dead-end hosts in the rabies transmission chain	Serengeti, Southeast Zone, Coast Zone

## Data Availability

No new data were created or analyzed in this study.

## Supplementary Materials

The following supporting information can be downloaded at: https://www.mdpi.com/article/10.3390/pathogens14090919/s1. Table S1: Preferred Reporting Items for Systematic reviews and Meta-Analyses extension for Scoping Reviews (PRISMA-ScR) Checklist; Table S2: Detailed list of the 20 studies identified through the database search and included in the review.
