# Supplementary material for: Rabies Surveillance in Mainland Tanzania: A Scoping Review of Animal Rabies Occurrences (1993–2023)
Source: Pathogens. 2025 Sep 11;14(9):919. doi: 10.3390/pathogens14090919 (PMC12472511; doi:10.3390/pathogens14090919)
Supplement: Supplementary file 1 [file pathogens-14-00919-s001.zip › pathogens-3764263-supplementary.pdf]

Supplementary Table S1: Preferred Reporting Items for Systematic reviews and Meta-Analyses extension for Scoping Reviews (PRISMA-ScR) Checklist.

SECTION	ITEM	PRISMA-ScR CHECKLIST ITEM	REPORTED ON PAGE #
**TITLE**	1	Rabies surveillance in mainland Tanzania: A scoping review of animal occurrences (1993–2023)	Page 1
**ABSTRACT**	2	Background: Rabies remains underreported in low-income countries, hindering control.<br>Objective: To synthesize data on animal rabies cases in Tanzania (1993–2023) and identify hotspots.<br>Eligibility Criteria: Peer-reviewed articles reporting animal rabies in mainland Tanzania.<br>Sources of Evidence: PubMed, Google Scholar, ScienceDirect.<br>Charting Methods: Screening done via Rayyan; descriptive synthesis used.<br>Results: 7,319 cases from 20 articles; most involved domestic dogs. Wildlife cases were rare but present.<br>Conclusions: Domestic dogs are key reservoirs; wildlife roles need further study. Enhanced surveillance is necessary in enzootic hotspots.	Page 3
**INTRODUCTION**			
**Rationale**	3	Due to underreporting, limited lab confirmation, and unclear wildlife roles in rabies spread in Tanzania, a scoping review is ideal to broadly map cases, identify hotspots, and highlight data gaps across species from 1993–2023.	Page 3,4 and 5
**Objectives**	4	-Estimate animal rabies cases in mainland Tanzania (1993–2023).<br><br>-To identify geographic hotspots of rabies transmission.	Page 3,4 and 5
**METHODS**			
**Protocol and registration**	5	No review protocol was registered for this scoping review.	"Not registered"
**Eligibility criteria**	6	Eligibility criteria: year (1993–2023), language (English), peer-reviewed only.	Page 5
**Information sources**	7	Information sources: PubMed, Google Scholar, Science Direct, searched on July 07, 2023, and September 12, 2023.	Page 5
**Search**	8	All 136 studies retrieved from PubMed are provided in the supplementary Excel file.	Supplementary file with included studies has been provided Supplementary File
**Selection of sources of evidence**	9	Screening was done using Rayyan; duplicates removed; title/abstract and full-text reviewed	Page 5

		using predefined inclusion/exclusion criteria; disagreements resolved by consensus.	
**Data charting process**	10	Data were charted using a standardized Excel form developed by the team. Charting was done independently by two reviewers, and discrepancies were resolved through discussion.	Page 6
**Data items**	11	Study title, author, year, location, animal species, diagnostic method, case count; assumed clinical diagnoses as probable when lab data missing..	Page 9,10 and 11
**Critical appraisal of individual sources of evidence**	12	Critical appraisal was not conducted, consistent with scoping review methodology.	Not applicable for scooping review
	13	Data were summarized using descriptive synthesis and tabulation, highlighting rabies cases by species, location, and time period.	Page 13 and 14
**RESULTS**			
**Selection of sources of evidence**	14	A total of 388 articles were screened; 20 met eligibility criteria and were included. Exclusions were due to duplication, lack of full text, or irrelevance. See PRISMA flow diagram.	Page 7 + Flow Diagram
**Characteristics of sources of evidence**	15	Data were charted on year of publication, study location, animal species involved, diagnostic method used, and number of reported rabies cases. Full citations are included in the reference list.	Page 13-14, Table 1
**Results of individual sources of evidence**	17	Data on species, case numbers, location, and diagnosis were extracted per study and summarized in a table.**	Xecel sheet suppliemtaRY attachment submitted
**Synthesis of results**	18	Charted data were summarized descriptively to highlight rabies occurrence, geographic hotspots, and species involved, addressing the review objectives.	Table 1
**DISCUSSION**			
**Summary of evidence**	19	Summary of main results, linking key themes and evidence to review objectives and their relevance.	Page 7–8
	20	Limitations include small sample sizes, limited species-level resolution, possible underreporting, and reliance on passive surveillance data which may affect comprehensiveness and accuracy.	Page 8
	21	Domestic dogs are main rabies reservoirs; hotspots identified. Enhanced surveillance and control needed. Supports elimination goals and further wildlife research	Page 8–9
**FUNDING**	22	Supported by AMED grants JP23wm0125008, JP243fa627005, and Hokkaido University junior scientist promotion project.	Page 10 or end of manuscript
